# Daily Cortisol Total Output Mediated Sleep and Affect Among Dementia Family Caregivers

**DOI:** 10.1093/geroni/igab046.681

**Published:** 2021-12-17

**Authors:** Yin Liu, Daniel Fleming, Elizabeth Fauth

**Affiliations:** Utah State University, Logan, Utah, United States

## Abstract

Cortisol is a primary stress hormone associated with sleep. We examined daily cortisol as the potential mechanism linking prior night’s sleep and daily mood among 173 dementia family caregivers (M (SD) age = 61.97 (10.66)) who used adult day services (ADS) at least two days a week. Caregivers self-reported sleep characteristics (bed and wake time, sleep quality, care receiver’s night-time problems) and affect (anxiety, depressive symptoms) across eight consecutive ADS/non-ADS days. Salivary cortisol was collected five times each day. Multilevel mediation analysis suggested that daily cortisol total output (assessed as “area under the curve”) mediated prior nights’ total time in bed and daily anxiety, but only on high-stress (non-ADS) days. Mediation was non-significant on low-stress (ADS) days, and at the between-person level. ADS use is respite from a chronically stressful role. Reducing exposure to stress via respite may protect against harmful processes related to sleep, cortisol reactivity, and daily anxiety.

